# The effect of naturally acquired rumen fluke infection on animal health and production in dairy and beef cattle in the UK

**DOI:** 10.3389/fvets.2022.968753

**Published:** 2022-08-18

**Authors:** Erwan Atcheson, Bernard Lagan, Ross McCormick, Hilary Edgar, Robert E. B. Hanna, Naomi H. Rutherford, Amanda McEvoy, Kathryn M. Huson, Alan Gordon, Aurelie Aubry, Mary Vickers, Mark W. Robinson, Jason P. Barley

**Affiliations:** ^1^School of Biological Sciences, Queen's University Belfast, 19 Chlorine Gardens, Belfast, Northern Ireland; ^2^Agri-Food and Biosciences Institute, Belfast, Northern Ireland; ^3^Dunbia, Dungannon, Northern Ireland; ^4^Agriculture and Horticulture Development Board, AHDB, Stoneleigh Park, Kenilworth, United Kingdom

**Keywords:** rumen fluke, paramphistome, *Calicophoron daubneyi*, dairy heifers, production, carcass

## Abstract

The incidence of paramphistomosis, caused by the rumen fluke, *Calicophoron daubneyi*, has greatly increased within Europe in the last 15–20 years. However, the production impacts of this disease are poorly understood. This study firstly aimed to investigate the prevalence of rumen fluke in England and Northern Ireland (NI) by conducting an abattoir survey of dairy and beef cattle which also allowed the impact of rumen fluke on carcass weight, conformation and fat classification to be assessed. Secondly, an experiment aimed to assess the impact of *C. daubneyi* infection on diarrhea score, production loss and welfare in dairy heifers, while also evaluating the impacts of treating infected heifers with oxyclozanide. Rumen fluke prevalence was greater in NI than in England, with 53.8% (95% CI 51.9 - 55.9%) of the NI cattle carcases sampled being infected compared to 16.3% (95% CI 15.8 - 16.8%) and 17.9% (95% CI 17.4 - 18.4%) detected at the two abattoirs in England. However, there was no significant difference (*P* > 0.05) in the cold carcass weight between infected and non-infected cattle. Similarly, carcass conformation and fat classification were unaffected (*P* > 0.05) by the presence of rumen fluke. In the second experiment, daily live weight gain (DLWG), diarrhea score and welfare score were also unaffected (*P* > 0.05) by rumen fluke infection and by oxyclozanide treatment against rumen fluke. The farms in this experiment were managed to a high standard and animals had no intercurrent disease. Therefore, these findings suggest that on well–managed farms, production losses (growth rates) should not be compromised as a result of sub-clinical rumen fluke infection.

## Introduction

Paramphistomes, also known as rumen flukes, are trematode parasites that infect ruminant livestock in tropical, sub-tropical and temperate regions worldwide (1, 2). Recent analyses of historical diagnostic records on the island of Ireland show a sudden and significant rise in the incidence of paramphistome infection which has been coupled with outbreaks of clinical disease and reported production loss (3, 4). Prior to 2009, prevalence of paramphistome eggs in Ireland averaged between 4 and 5% (3). Over the period 2009 to 2013 the proportion of bovine fecal samples positive for paramphistome eggs increased to a peak of 32% in 2010, followed by lower prevalence of 20% in 2011 and 2012 and a further peak of 28% in 2013. Ovine samples showed a similar but more dramatic increase with a peak prevalence of around 50% in 2011 (3). Based largely on fecal egg counts, rumen fluke infection has also steadily increased across the UK since its emergence in England, Wales and Scotland in 2007 (5, 6). For example, in the 60 months period January 2007 to January 2012, nine diagnoses of paramphistome infection were made in Scotland and 188 in England and Wales. Whilst in the 12 months period January 2012 to January 2013, 20 diagnoses were recorded in Scotland and 119 in England and Wales (5). The reasons for this increase in incidence of paramphistome infection in United Kingdom and Ireland are not fully understood. The situation may reflect the introduction of a new species or the expansion of pre-existing populations due to climatic effects. *Calicophoron daubneyi* is the most common rumen fluke in Scotland and possibly the only rumen fluke present in Ireland (3, 6). Previous reports that *Paramphistomum cervi* was the rumen fluke species prevalent in Great Britain now appear to be inaccurate (6). *C. daubneyi* shares the *Galba truncatula* snail host with the liver fluke *Fasciola hepatica* although the literature would indicate that co-infection of both species is unlikely, due to interspecies predation, competition for nutrients and changing biochemical composition of snail tissue (7–9).

Current prevalence of *C. daubneyi* infection in cattle herds in Ireland is high. Summer grazed / winter housed beef suckler and dairy herds show a rising prevalence of infection during the spring and early summer to a peak during June to September when between 57 and 100% of fecal samples from individual animals show the presence of paramphistome eggs. Prevalence then falls during the autumn and winter months (3). Reasons for the autumn decline in populations are not well–understood but investigations of paramphistomosis in Spain have shown variable overall prevalence of infection which appears to be region-dependent and influenced by management practices, stocking rate, age (10) and various environmental effects such as soil drainage, land cover and habitat, rainfall and vegetation (11). Modeling has shown that the presence and prevalence of *C. daubneyi* was positively associated with higher summer rainfall and/or rain days (12). Infection rates may increase with increasing rainfall up to the point of removal of snail habitats or separation of miracidia from snail hosts by flooding – the so called “wash out effect” (13). Equally, gently sloping land may increase the probability of infection due to a swamping effect and increase in size of snail habitats (14).

Sub-clinical and chronic paramphistomosis involving mature rumen flukes (of species other than *C*. *daubneyi*) is considered a production-limiting disease in many tropical and sub-tropical regions (15). The economic losses associated with paramphistomosis in these cattle production systems are often due to a heavy burden of mature rumen flukes, which may negatively impact production traits like feed intake, carcass weight, carcass conformation, growth rate and milk yield (16, 17). In Europe it remains unclear whether chronic infection significantly impacts cattle productivity or welfare although adult paramphistomes are generally believed to be well–tolerated by the host, even when present in high numbers (2, 18). However, a histological study conducted by Fuertes, Pérez (19) described severe tissue damage including inflammation and ulceration at the site of *C. daubneyi* fluke attachment. Similar reports have also been described for livestock animals infected by the tropical paramphistomes *Paramphistomum ichikawai* and *Calicophoron microbothrium* (16, 20). This raises questions regarding the veracity of this “well-tolerated” status for mature rumen flukes. For example, De Waal (21) reported that acute rumen fluke infection could be characterized by listlessness, anorexia and diarrhea. Furthermore, an abattoir study described a negative association between adult rumen fluke and the cold carcass weight and fat classification of British cattle (22). While this finding is limited by a small sample size, it highlights the need for more comprehensive studies detailing the potential negative effects of this emerging disease to the cattle industry.

In this study we conducted an abattoir survey to estimate the prevalence and severity of *C. daubneyi* infection in beef and dairy cattle in the UK and to identify any correlation with carcass weight and classification. Furthermore, in an attempt to support existing anecdotal evidence of diarrhea, adverse welfare and production loss due to rumen fluke, we performed the first experimental field trial to investigate the effect of *C. daubneyi* infection on these parameters in dairy heifers in the UK. Our study presents a more precise definition of the effects of paramphistomosis (other than acute clinical disease) which is important to drive treatment decisions and control programmes for this infection.

## Materials and methods

### Study one: Abattoir survey

**Figure 1:**
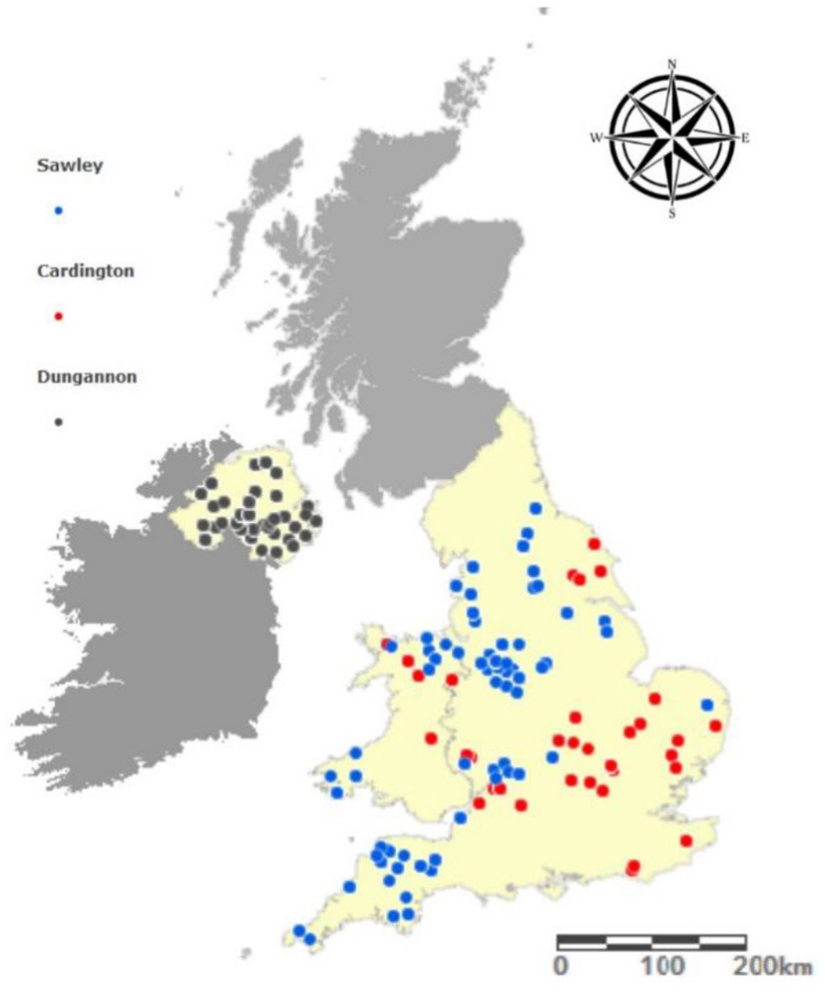
The geographic origin of cattle carcases sampled from each of the abattoir sites.

During a 3 months period from November 2018 to January 2019 a survey was conducted across three Dunbia Ltd. abattoirs, two of which were located in England (Cardington and Sawley), and the third was located in Northern Ireland (Dungannon) (Figure 1). On each sampling day, all cattle carcasses on the kill line were included in the study, therefore the data recorder had no impact on which carcasses were presented. A total of 1,910 carcasses were examined over the 3 months period.

#### Abattoir measurements

Beef carcass classification is required under European Union (EU) legislation (EC Regulation 1184/2017) and links a unique kill number to the following demographic and carcass information: date of slaughter, farm origin (postcode), breed, sex, category (cow, heifer, steer, young bull, stock bull), production system (dairy or beef) and cold carcass weight (CCW in kg). Additionally, carcass conformation is graded according to the categorical EUROP scale, ranging from E (excellent carcass conformation) to P (poor carcass conformation). Fat classification is scored from 1 to 5 to evaluate the external fat cover of the carcass where 1 represents very lean and 5 represents very fatty. Carcases assessed at Cardington and Sawley (England) were manually graded by a meat inspector using the 5-point grid, whereas in Dungannon (Northern Ireland) Video Image Analysis (VIA) machines were used to inspect and grade the carcases using a 15-point grid. To decipher the geographic location of the farms the carcases originated from, postcodes were converted to latitude and longitude (www.gridreferencefinder.com) and mapped using ArcGIS Online (ArcGIS^®^ software by Esri). The slaughter-age was calculated in months by subtracting the date of birth from the date of slaughter.

Reticulorumens and livers from cattle were randomly selected and examined at slaughter. The internal mucosa and reticular groove of the rumens were visually examined for the presence or absence of adult rumen fluke and scored on a numerical 6 point scale of 0–5 (0 = <1 flukes; 1 = ≥1 flukes <50; 2 = ≥50 flukes <200; 3 = ≥200 flukes <500; 4 = ≥500 flukes <1,000; 5 = ≥1,000 flukes). Livers were inspected as per the Food Standards Agency's (United Kingdom) Meat Hygiene Service for evidence of fasciolosis and scored based on the presence or absence of *F. hepatica* and/or hepatic lesion severity due to liver fluke on a 2 point scale (0 = neither adult *F. hepatica* nor liver fluke lesions present; 1 = adult *F. hepatica* and/or liver fluke lesions present).

#### Statistical analysis

##### Prevalence and severity of fluke infections

Prevalence percentages for mature rumen fluke, liver fluke and co-infection were calculated for each abattoir site. A chi-square test was used to compare rumen fluke prevalence between Cardington, Dungannon and Sawley. The burden of rumen fluke infection was mapped to farm origin using the scoring system described above with ArcGIS Online (ArcGIS® software by Esri).

To investigate co-infection, the expected rate was calculated assuming that the occurrence of rumen fluke and liver fluke infection were independent of each another. Chi-square analysis was then used to determine whether the difference between observed and expected rate of co-infection was statistically significant. Odds ratio was used to investigate whether the rate of adult rumen fluke infection in cattle was associated with the presence of *F. hepatica* or liver fluke damage.

Because of high prevalence levels at Dungannon compared to the other sites, the next series of analyses were restricted to data from Dungannon. Specifically, Fisher's exact test was used to investigate whether rumen fluke prevalence differed between production systems, breed, sex and/or age (<24 months, 24–30 months, >30 months).

##### Impact of rumen fluke infection on carcass parameters

Multilevel mixed-effect linear regression was used to model the effect of rumen fluke infection on three carcass parameters: cold carcass weight, carcass conformation grade and fat class. Analysis was restricted to cattle reared for prime beef: heifers, steers and young bulls aged 12–36 months. In each model, breed category, age, month of abattoir collection, and helminth infection (rumen fluke or liver fluke together or alone) were modeled as fixed effects, and herd (by postcode) as random effects, fitted in R using the lme4 package and the restricted maximum likelihood method.

##### Carcass and economic parameters

In Dungannon the association between fluke infection and carcass parameters of the five most frequent breeds were assessed (Charolais, Friesian, Hereford, Holstein, and Limousin). Unpaired *T* tests with Welch's correction was used to examine the association between cold carcass weight (kg) and infection status (no fluke infection, rumen fluke only, *F. hepatica* only, co-infected), independently accounting for breed, age and sex. A Mann-Whitney-Wilcoxon test was used to analyse the fatness and conformation score of cattle infected and not infected with rumen fluke and/or liver fluke by converting into a numerical scale described by Pritchard, Wall (23).

To evaluate the carcass performance and market demand for the cattle assessed at the Dungannon abattoir, the date of birth, slaughter date, breed, sex category, carcass weight and carcass grade were placed into the Agriculture and Horticulture Development Board (AHDB) beef carcass calculator (https://ahdb.org.uk/beef-and-lamb-carcase-calculators). This approach may not apply to all market conditions, because carcass market value will vary depending on region and time of year. It is a first attempt to quantify the effect of infection on product value. Fisher's exact test was used to evaluate if there was any significant difference between cattle infected and not infected with rumen fluke.

### Study two: Dairy heifer field trial

#### Experimental design

Four commercial dairy farms [Sites (S) 1–4], two in Northern Ireland (S1 & 2) and two in South West England (S3 & 4) were selected based on pre-trial health records showing confirmed rumen fluke infection within the herd. A herd was considered infected if a pooled fecal sample showing ++++ or >100 eggs per gram (epg) was obtained on multiple occasions from adult dairy cows and/or dairy young stock. The study was conducted from April 2017 to mid-August 2019. During the study all animals were at pasture throughout the main grazing season and then housed indoors during winter. The field study involved two phases which are outlined below.

#### Phase 1 experimental design (first grazing season)

Phase 1 was conducted during the first grazing season of the study and examined the effect of rumen fluke infection on the diarrhea score, welfare assessment and live weight gain of dairy heifers at all four sites. It involved a minimum of 50 first season grazing (born in spring 2017) Holstein x Friesian heifers from each site (S1 *n* = 57; S2 *n* = 52; S3 *n* = 50; S4 *n* = 50), which were selected prior to turnout to grass. A power calculation indicated that given the expected variation in the data, a minimum sample size of 20 would be required to detect a slope of 0.01 (or greater) as being significantly different from 0 at the 5% significance level with 80% power. Proprietary randomization software was used to select study animals from the first season heifers on the farm for phase 1.

A herd health plan approach was used to control coccidiosis, gut worm, liver fluke and lung worm infection in all of the study animals. It is important to note that products containing either oxyclozanide or closantel were not used for the control of liver fluke because of the therapeutic effect of these compounds on rumen fluke.

#### Phase 2 experimental design (second grazing season)

Phase 2 was conducted during the second grazing season of the study and investigated the effect of treatment of rumen fluke infection with oxyclozanide on the diarrhea score, FEC, welfare assessment and bodyweight of dairy heifers. Phase 2 consisted of two treatment groups; (i) rumen fluke treated or (ii) rumen fluke untreated. The rumen fluke treated group were orally dosed with Zanil Fluke Drench 34 mg/ml (oxyclozanide: MSD Animal Health) on three occasions (i) during the early part of the grazing season (May 2018), (ii) when pooled FECs first showed the presence of rumen fluke infection and (iii) at housing in November 2018. The same herd health plan outlined in phase 1 was implemented to control coccidiosis, gut worm, liver fluke and lung worm infection in all of the study animals. Phase 2 was conducted on sites S1 and S2 only, due to the significant rumen fluke infection on these sites. As this study was conducted the following year, the same animals from phase 1 were utilized as rumen fluke infected second season grazers and proprietary randomization software was used to allocate to treated or untreated groups. In addition, a further group of first season grazing heifers (*n* = 67) on S1 were included with random assignment of half the heifers to each treatment group. These latter heifers were included in the study based on statistical advice that if infection occurred in the group as expected, useful data on the effect of removal of rumen fluke by oxyclozanide treatment could be obtained. The two treatment groups were grazed in separate paddocks within the same pasture or shared pasture if this was not possible.

#### Measurements

In both phase 1 and 2 all animals were weighed, diarrhea scored, had fecal samples taken for pooled FEC and were welfare assessed. All measurements were taken at monthly intervals from April through to November. Daily live weight gain was calculated using the start weight and end weight for each phase.

**Figure 2:**
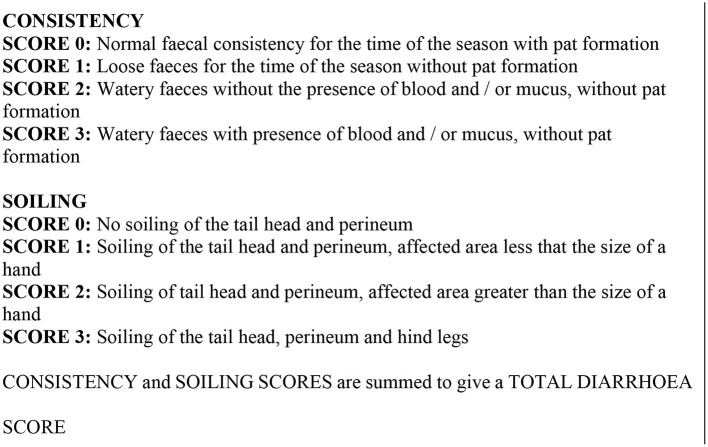
Diarrhea scoring system.

The diarrhea scoring system was a composite of fecal consistency and degree of soiling to give a total diarrhea score as shown in Figure 2. Approximately 10g of feces was collected from each animal at each monthly sampling occasion and individual samples from 10 animals were used to form a pool for testing in phase 1. On each sampling occasion the same 10 animals were used to provide a pooled sample. In phase 2, counts were carried out using similar methodology but on individual samples. Three grams of feces from each pool were diluted with 420 ml of water and aliquoted into three test tubes for FEC for nematode eggs using the McMaster method (24) and for liver fluke and rumen fluke eggs by sedimentation (25). The individual counts of each of the three samples derived from a single pool were averaged to give the final count for that pool.

Welfare was assessed based on the good health principle of animal welfare according to the absence of disease criterion, using total diarrhea score and live weight gain as the primary criteria but also utilizing general health data recorded for study animals. Complete health records were kept for each of the study animals, diagnosis of disease, treatments and outcomes including withdrawal from the trial were recorded and these formed a part of the assessment of the effect of rumen fluke on animal welfare.

#### Statistical analyses

Proprietary randomization software was used to select study animals from the first season heifers for phase 1 and to allocate study animals to rumen fluke treated and untreated treatment groups during phase 2. In phase 1 the experimental unit was the pool of 10 animals used to make up a sample pool for rumen fluke larvae and FEC whilst in phase 2 the experimental unit was the individual animal. In phase 2, the identity of the rumen fluke treated and untreated groups were unknown to the investigator carrying out the diarrhea scoring and welfare assessment.

In phase 1, because the measurement occasions differed across farms, the diarrhea scores and FECs for each pool were averaged across all measurement occasions. Daily live weight gain across the study period was calculated for each individual animal and then averaged over the 10 animals in each pool. The resultant variables were then analyzed using a one-way ANOVA to assess if there was any significant difference between farms. If this was the case, Fisher's Least Significant Difference Test was used to check for pairwise differences between farms. In addition a linear contrast was used to see if there was any difference between fluke infected and non-fluke infected farms.

In phase 2 individual animals were measured over the year on a number of occasions and were also analyzed on this basis. Egg counts were analyzed using generalized linear mixed model methodology using the REML estimation method with a Poisson distribution and logarithm link function. Farm and animal within farm were fitted as random effects in the model while treatment was fitted as a fixed effect. Diarrhea score was analyzed using ordinal logistic regression with random effects (proportional odds model) with the same fixed and random effects. The daily live weight for each animal was calculated across the study period and subsequently analyzed as a linear mixed model using the REML estimation method with farm fitted as a random effect and treatment as a fixed effect.

All analyses were carried out in GenStat 19.1 except for the diarrhea score in phase 2 with was carried out using Stata 14.2.

## Results

### Study one: Abattoir survey

#### Description of abattoir and animal parameters

A total of 1,910 completed animal records (i.e., known fluke status and no missing covariates) were included in this study: 650 (34 %) in November, 620 (32 %) in December, and 640 (34 %) in January. Of these carcases, 607, 609, and 694 were sampled in Sawley, Cardington, and Dungannon respectively. 137 farms were identified to geographical origin, with 26% (35/137) in Northern Ireland, 11% (15/137) in Wales, and 63% (87/137) in England. There were 1,010 (53%) male cattle and 900 (47%) female cattle included in this study, of which 50% (948/1,910) were steers, 29% (546/1,910) were heifers, 19% (353/1,910) were cows, 3% (59/1,910) were young bulls and <1% (3/1,910) were stock bulls. A total of 70 breeds were included: 41% (779/1,910) were from beef pure breeds, 27% (509/1,910) from beef cross breeds, 26% (501/1,910) from dairy pure breeds, 4.5% from dairy cross breeds (88/1,910) and 1.5% (33/1,910) from multi-purpose breeds. Table 1 shows the overall demographic, weight and carcass classification of the cattle in the Dungannon abattoir dataset.

**Table 1:** The median [p25–75] age, cold carcass weight, carcass conformation and fat classification score for each category of cattle included in the Dungannon abattoir dataset excluding mature bulls (*N* = 691).

**Variables (*N*)**	**Cows (140)**	**Heifers (180)**	**Steers (324)**	**Young bulls (47)**
Age (months)	72 [53–98]	27 [24–30]	29 [24–39]	15 [14–15]
Cold carcass weight (kg)	302 [268–328]	323 [294–347]	342 [303–380]	317 [296–317]
Carcass conformation	P^+^ [P-O]	R^−^ [O-R^+^]	O^+^ [O^−^ -R]	O^+^ [O^−^ -R^+^]
Fat class	3 [2^+^ −4^−^]	3^+^ [3–4]	3 [3^−^−3^+^]	3^−^ [3^−^−3^−^]

#### Prevalence and severity of rumen fluke infections

**Figure 3:**
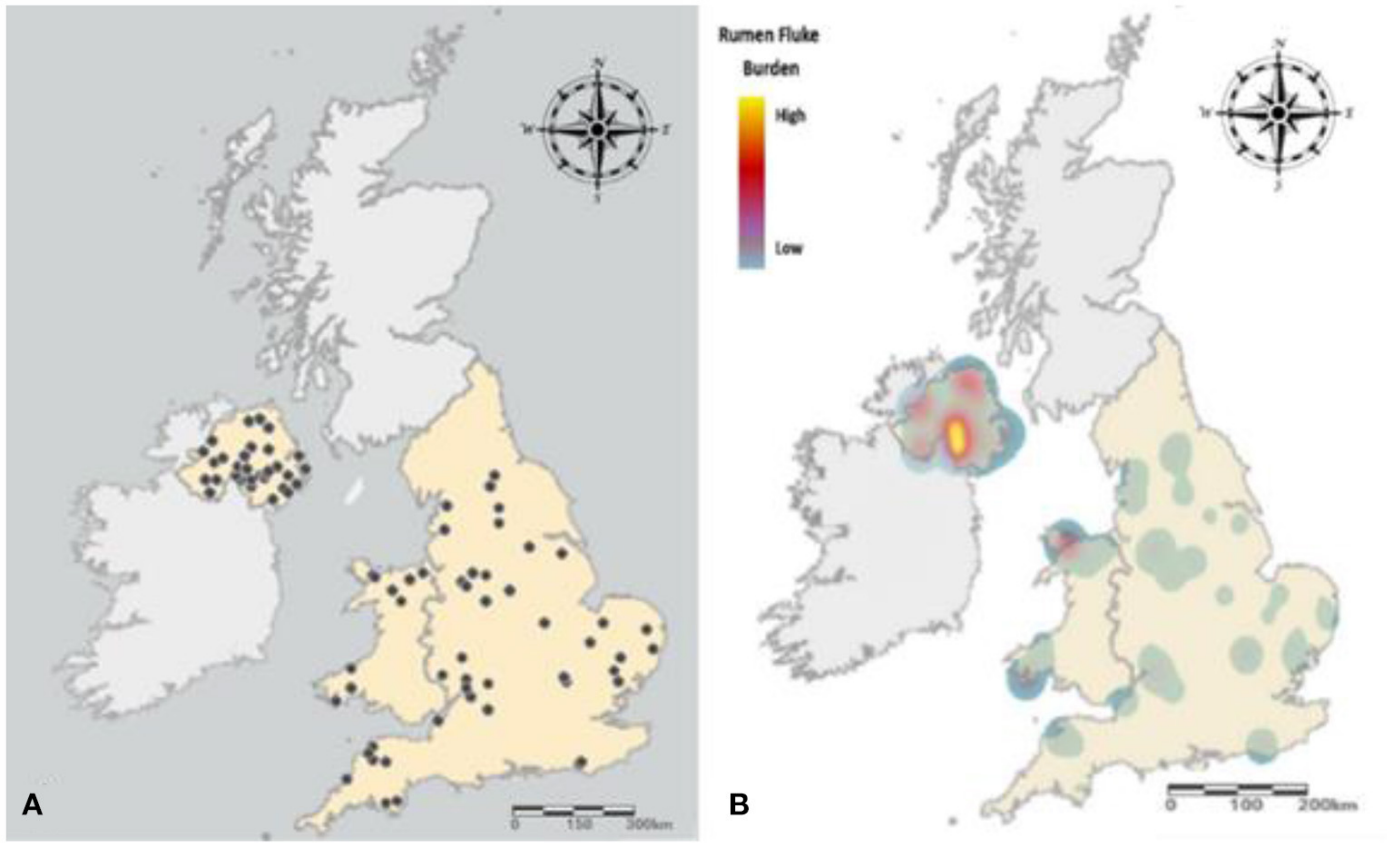
Distribution and prevalence of cattle carcases positive for rumen fluke. **(A)** Farm origin of cattle carcases infected with rumen fluke. **(B)** The prevalence and burden of rumen fluke infection experienced across England, Wales and Northern Ireland. A low burden (<50 flukes) is represented by blue, with the rise in color gradient synonymous with an increase in rumen fluke burden, with a very heavy burden (>1,000) represented by yellow.

No significant difference was observed between the prevalence of rumen fluke infection between November, December or January (*P* = 0.9341). However, rumen fluke prevalence was significantly higher in Northern Ireland, with 53.8% (95% CI 51.9 - 55.9%) of cattle carcases sampled in Dungannon infected compared to 16.3% (95% CI 15.8 - 16.8%) in Cardington and 17.9% (95% CI 17.4 - 18.4%) in Sawley (χ^2^ = 142.3, *P* = < 0.0001) (Table 2). Farm-level prevalence ranged from 89% (31/35) in Dungannon to 47% in both English sites (Cardington 17/36; Sawley 32/68). Northern Ireland also experienced a heavier burden of rumen fluke, with 9.5% (66/694) of reticulorumens inspected containing over 500 adult flukes, compared to 1.3% (16/1,216) inspected in the English abattoirs (Fisher's exact test, *P* = < 0.0001) (Figure 3).

**Table 2:** The prevalence of adult rumen fluke, liver fluke or both in cattle carcasses from three abattoir sites in the United Kingdom.

	**Abattoir site**		
	**Cardington**	**Dungannon**	**Sawley**
Total carcases sampled	607	694	609
Rumen fluke prevalence (%)	16.3	53.8	17.9
Liver fluke prevalence (%)	4.8	26.2	9.7
Expected co-infection prevalence (%)	0.8	14.0	1.7
Observed co-infection prevalence (%)	1.7	14.6	2.0
Ratio observed: expected co-infection prevalence	2.1	1.0	1.2
Chi-square value	5.042	0.192	0.407
*P* value	0.025	0.66	0.52

*The expected rate of co-infection was calculated for each site with the assumption that the occurrence of both helminth parasites was independent of one another. Chi-square analysis was used to determine if there was a significant difference between the observed to expected ratio for co-infection at each site. Significance was declared at P < 0.05*.

#### Occurrence of co-infection

Co-infection rates at Dungannon (14.6%) were substantially greater than the other sites (Table 3). Of the animals infected with rumen fluke, 27% (101/374) were co-infected with *F. hepatica* in Dungannon, compared to 11% (10/99; 12/109) in Cardington and Sawley. However, Cardington was the only site at which the joint occurrence of rumen fluke and *F. hepatica* was significantly higher than the expected value (χ^2^ = 5.042, *P* = 0.0247) (Table 2). In addition, the odds of cattle sampled from Cardington being infected with rumen fluke were nearly three times higher (Odds Ratio 2.892, 95% CI 1.30 to 6.43) in cattle infected with *F. hepatica* than in cattle free from *F. hepatica* (χ^2^ = 7.369, *P* = 0.0066). In contrast, cattle carcases sampled from Dungannon and Sawley abattoirs showed no significant association between the presence of *F. hepatica* and an increased susceptibility to rumen fluke infection. Within Dungannon, the highest prevalence of co-infection was recorded in cows, with 34.5% (20/58) of carcases infected with rumen fluke also presenting signs of *F. hepatica* infection, with 29.7% (30/101) for heifers, 25.8% (48/186) for steers and 11.1% (3/27) for young bulls.

**Table 3:** Effect of rumen fluke infection on three measures of carcass quality.

**Fixed effects**			**Model 1: Cold carcass weight**			**Model 2: Carcass conformation**			**Model 3: Fat classification**		
Variables	Categories	N	Beta	95% C.I.		Beta	95% C.I.		Beta	95% C.I.	
Intercept (SE)			306.109	4.134		6.810	0.157		9.425	0.160	
Helminth infection	None	873	*Baseline*								
	RF only	330	−1.682	−7.333	3.969	−0.155	−0.379	0.070	−0.098	−0.331	0.135
	LF only	115	4.304	−3.791	12.399	0.179	−0.143	0.502	0.161	−0.174	0.496
	RF & LF	87	−4.784	−14.351	4.783	−0.082	−0.463	0.298	−0.080	−0.476	0.316
Random effects	Level		Variance			Variance			Variance		
	Herd		696.6			0.8507			0.8239		
	Cattle		292.5			0.6863			0.8091		

*RF, Rumen fluke; LF, Liver fluke*.

#### Animal level prevalence of rumen fluke infection

**Figure 4:**
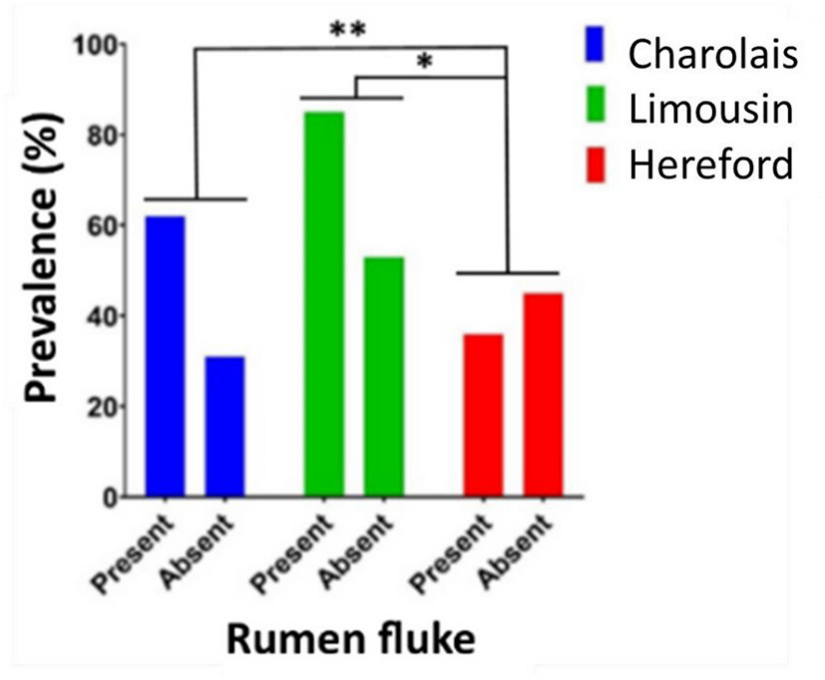
The prevalence of rumen fluke infection in beef cattle carcases. The prevalence percentage of cattle carcases infected with (present) or not infected with (absent) rumen fluke sampled from Dungannon abattoir for the three most frequent beef breeds in this study evaluated by Fisher's exact test; *P* < 0.05*; *P* < 0.01**.

In Dungannon the beef production system was more susceptible to rumen fluke infection than the dairy production system (Fisher's exact test, *P* = 0.0477). However, rumen fluke prevalence was high in both, ranging from 40% in Holstein dairy cows (16/40) to 67% in Charolais beef cattle (62/93). Individual assessment of cattle breeds found that the continental beef breeds, Charolais and Limousin, had a significantly higher prevalence of rumen fluke infection when compared to British Hereford beef cattle (Fisher's exact test: Charolais and Hereford *P* = 0.0037; Limousin and Hereford *P* = 0.0168) (Figure 4). Within each breed investigated, there was no statistical difference between age category or sex with regards to rumen fluke prevalence.

#### Implications of fluke infection on carcass and economic parameters

In Dungannon there was an insufficient number of animals with high levels of rumen fluke (>500 rumen fluke; *n* = 66) present in the reticulorumen to allow for statistically validated evaluation of the effects of rumen fluke burden on carcass parameters given that breed, age and sex have to be accounted for. Consequently all inferential analysis of rumen fluke infection was based on a present or absent basis.

**Figure 5:**
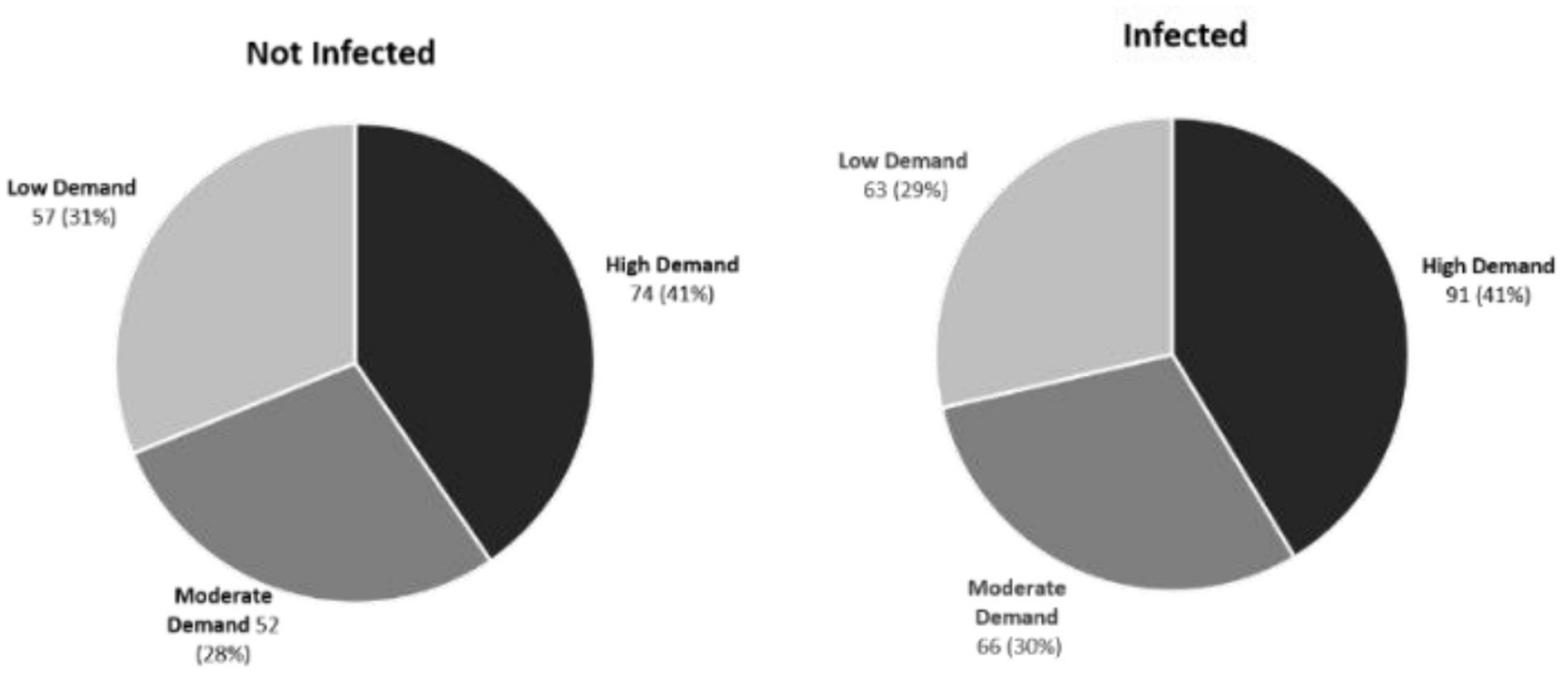
Market demand for cattle carcases infected or not infected with rumen fluke. No significant difference between the high, medium or low market demand for carcasses sampled from the Dungannon abattoir was demonstrated based on the presence or absence of rumen fluke infection.

Using the abattoir data, relationships between rumen fluke infection and three carcass parameters (cold carcass weight, carcass conformation and fat class) were modeled using multilevel mixed-effect linear regression. Although rumen fluke infection correlated negatively with all three carcass parameters, this effect was never statistically significant (Table 3). Using the AHDB carcass calculator, adjusting for breed, age, carcass grade, carcass weight and sex category, it was revealed that there was no significant difference between the market demand for the carcases sampled in Dungannon abattoir (Fisher's Exact Test; *P* = 0.9042) (Figure 5).

### Study two: Dairy heifer field trial

#### Phase 1

In phase 1 there was no significant difference (*P* > 0.05) in diarrhea score or DLWG between infected and non-infected farms (Table 4). On the infected farms, pooled FEC was significantly greater (P < 0.001) on S1 (19.9 ± 2.04 epg) than on S2 (3.3 ± 2.04 epg). Welfare assessments indicated no adverse welfare due to rumen fluke infection on any of the four sites on the basis of recording diarrhea score, growth rate, significant clinical disease or mortality and the application of the good health principle.

**Table 4:** Diarrhea score, daily live weight gain and pooled rumen fluke fecal egg counts.

**Variable**	**S1**	**S2**	**S3**	**S4**	**SEM**	**Prob**.
Diarrhea score	0.989	0.868	0.648	1.408	0.0782	0.246^a^
DLWG (kg/d)	0.919	1.000	0.843	1.001	0.0366	0.370^a^
FEC (epg)	19.9	3.3			2.04	<0.001

a *Probability relates to comparison between infected (S1 & S2) and non-infected (S3 & S4) farms*.

*DLWG, daily live weight gain; FEC, fecal egg count*.

#### Phase 2

Table 5 shows that the treatment of rumen fluke in dairy heifers had no significant impact (*P* > 0.05) on DLWG. Yet FEC was significantly (*P* < 0.001) reduced when heifers were treated with oxyclozanide. As shown in Table 6 diarrhea score did not differ between treated and untreated heifers. Similar to phase 1, no adverse welfare due to rumen fluke infection was recorded on either of the two sites.

**Table 5:** Daily live weight gain and fecal egg count of treated and untreated groups on S1 and S2.

**Variable**	**Treated**	**Untreated**	**SED**	**Prob**.
FEC (epg)	11.28 (0.771-165.1)^a^	35.52 (2.42-521.2)^a^	-	<0.001
DLWG (kg/d)	0.93	0.94	0.023	0.514

a *Predicted count with 95% confidence intervals*.

*FEC, fecal egg count; DLWG, daily live weight gain*.

**Table 6:** Diarrhea Score of treated and untreated groups on S1 and S2.

**Diarrhea score**	**Treated**	**Untreated**	**Prob**.
0	0.570 (0.371–0.769)^a^	0.627 (0.435–0.820)^a^	0.074
1	0.261 (0.176–0.346)^a^	0.237 (0.146–0.328)^a^	
2	0.130 (0.040–0.219)^a^	0.106 (0.025–0.187)^a^	
3–4	0.039 (0.000–0.084)^a^	0.029 (0.000–0.064)^a^	

a *Predicted probabilities with 95% confidence intervals*.

## Discussion

### Study one: Abattoir survey

#### Prevalence and burden of rumen fluke

This abattoir study demonstrates the variation of rumen fluke prevalence exhibited at a national scale throughout the United Kingdom. In Northern Ireland the overall abattoir prevalence of 53.8% is significantly higher than that reported from the English abattoirs of 16.3 and 17.9%. While this is the first study conducted that has evaluated rumen fluke prevalence in Northern Ireland, the high level of bovine paramphistomosis is very similar to that of the Republic of Ireland, where 52% of cattle were infected (26). In contrast, the cattle carcases investigated from England and Wales demonstrated a lower level of rumen fluke infection when compared to the abattoir prevalence rate of 25 to 29% previously reported for the United Kingdom (22, 27). This can be observed in the widespread distribution of rumen fluke in Northern Ireland, where the farm-level prevalence of 88.6% is likely the result of high levels of rainfall and overall habitat suitability that supports the transmission of infection between the molluscan intermediate hosts and the definitive hosts (11). Furthermore, the lower prevalence observed in England may be a result of dry weather conditions in summer 2018 illustrated by a 36% lower total rainfall from June to September than that of Northern Ireland (28, 29). For example, Titi, Mekroud (30) found that *C. daubneyi* infection significantly decreased in young bulls following extreme drought in Algeria.

A significantly greater proportion of cattle from Northern Ireland were heavily infected with over 500 flukes when compared to England and Wales. However, the overall median burden for this current study was low, with most infected cattle inspected at each of the abattoir sites containing <50 adult rumen flukes. This is concordant with reports from the Republic of Ireland (26) and France (31), but unlike those from Spain where the median burdens have been demonstrated to exceed 250 rumen flukes (13, 32). Unfortunately given the limited sample size of cattle with heavy burdens included in this study (*n* = 66), it was not possible to exclusively assess the impact of severe rumen fluke burden on cattle performance as there were too many independent variables to consider (e.g., breed, sex, category, age and production system).

#### Impact of fluke infection on cattle carcass performance

The overall demographic, weight and carcass classification described for the population of cattle sampled from the Dungannon abattoir (Table 1) were consistent with a previous survey evaluating the British beef production industry (23). As this industry is driven by market demands, farmers often present cattle for slaughter which adhere to target specifications to ensure better financial returns (33). In this study, beef cattle and dairy cull cows infected with rumen fluke demonstrated no significant decrease in carcass performance when compared to uninfected animals. Similar findings were reported by Sargison, Francis (27) who investigated rumen fluke infections in prime beef cattle. In contrast, Bellet, Green (22) described a significant reduction in weight and fat deposition in cattle carcases harboring single and co-infections of rumen fluke with *F. hepatica* and species belonging to the gastrointestinal (GI) nematode genus *Ostertagia*. Although there was no significant association between co-infection with *F. hepatica* and carcass parameters in this current study, the presence of GI nematodes would also need to be considered. These were not recorded in the present abattoir dataset. Concordant with the findings presented by Bellet, Green (22), Charlier, De Cat (34) demonstrated that *O. ostertagi* negatively impacts the conformation score and carcass weight of beef cattle. With a reported prevalence of 89 to 100% in cattle livestock from the United Kingdom and Ireland, it is likely that the animals uninfected by flukes in this study may have harbored GI nematode infections (22, 35). Furthermore, of the carcasses that were co-infected with rumen fluke and *F. hepatica*, it was difficult to discriminate between the compounding effects of this dual infection. Further studies, using experimentally-infected animals, would help to unravel this (36).

Cost-benefit analysis is valuable to livestock producers to justify investments into disease control. Given the limited knowledge surrounding the biology, epidemiology and pathogenicity of rumen fluke within the United Kingdom, it is difficult to establish the economic impact of this emerging parasite at a farm and national level. In this current study, it appears that the presence of rumen fluke does not affect the market demand for beef cattle sampled in Northern Ireland (Figure 5). However, this does not account for the burden of rumen fluke within each cattle carcass. In order to get a true estimation of the financial impact of rumen fluke to the cattle industry it is imperative that a greater understanding of heavy infestations during chronic paramphistomosis is acquired. Although a coproantigen test for *C. daubneyi* was recently described (37), novel diagnostic methods demonstrating a high sensitivity for the detection of immature *C. daubneyi* are required to help evaluate the economic significance of acute paramphistomosis which often has more significant pathology (38, 39).

### Study two: Dairy heifer field trial

#### Phase 1

Our findings are in agreement with Malrait, Verschave (40) who found no association between rumen fluke infection and fecal consistency at the herd level. However, Malrait, Verschave (40) used adult dairy cows, which are less likely to display signs of diarrhea than younger cattle (39). Thus, as suspected by Malrait, Verschave (40), it is possible that infection levels were insufficient to result in clinical signs of rumen fluke, which may also have been the case in our study as indicated by the FECs shown in Table 5. This is further supported by the findings of Mavenyengwa, Mukaratirwa (41), where experimentally infected cattle only displayed signs of diarrhea after being infected with medium or high levels of *Calicophoron microbothrium* metacercariae. Furthermore these clinical signs were short lived; developing 21 days post-infection and only persisting for 3 days. However, it should be noted that these authors (40, 41) examined different species of rumen fluke and thus this may account for some of the variation observed. Foster, Otter (42) reported on five cases that had been submitted to the Veterinary Laboratories Agency (VLA). The five cases were all adult cows and diarrhea was the most common symptom stated. Large numbers of rumen fluke eggs were present in all five fecal samples, and four samples also contained liver fluke eggs. Thus, the high level of co-infection may also impact on the severity of clinical symptoms.

The impact of rumen fluke on the DLWG of ruminants has received little attention within the literature. In lambs, natural infection by *C. daubneyi* has been deemed to have no marked impact on DLWG (43); which supports the findings in the current study. Loss of condition has been reported in infected cattle (44) and could be caused by inflammatory reactions in the rumen and reticulum (19). The developmental stage of rumen fluke is thought to contribute to the clinical signs of disease that are exhibited by infected animals, with greater clinical infection being associated with immature flukes (38, 39), whereas adult flukes are better tolerated, even in large numbers (39, 45). As our infection rates were determined by FEC, this only gives an estimation of the adult flukes present, therefore, the severity of the burden of immature flukes was unknown (32).

#### Phase 2

Oxyclozanide treatment significantly reduced paramphistome FECs (68.2%) however this did not occur to the magnitude that would have been expected. Previous research has reported a reduced egg-output of 98–99% in cattle naturally infected with *C. daubneyi* (32). This indication that oxyclozanide may have limited efficacy under normal grazing conditions in the United Kingdom is of concern. In addition to the considerable cost to animal producers, ineffective oxyclozanide treatment may actually select for resistance in liver fluke populations meaning that an effective adulticide may also become of limited use against liver fluke.

This study shows no evidence of a difference in either diarrhea score or DLWG between treated and untreated groups. Furthermore, no adverse impact on animal welfare due to rumen fluke infection was recorded on either of the two sites. We are of the opinion that this is an accurate representation rather than a deficiency in the partially subjective diarrhea scoring system because there were differences between farms in fecal consistency albeit not related to rumen fluke infection. Treatment with oxyclozanide has previously been associated with clinical improvement (42). However, in the report by Foster, Otter (42) infected cattle were displaying moderate signs of ill health, which was not the case in our study.

Previous studies have highlighted the effectiveness of oxyclozanide on adult and immature rumen flukes (32, 46–48). However, the presence of immature rumen fluke was not assessed in this study and therefore they may still have been present and undetected within these animals (49). Although, even if this was the case, these animals showed no signs of compromised welfare, and DLWG was of a high standard and similar to that of previous research (50).

In short, this field study does not support the anecdotal evidence of adverse production effect due to rumen fluke infection and we did not see any acute infection (due to immature intestinal-stage flukes) which would have altered the picture. We are of the opinion that these findings can be applied only to well-managed, healthy dairy heifers. The effect of rumen fluke infection on cattle compromised by intercurrent disease could not be assessed as a part of this study.

## Conclusion

Abattoir studies have shown that Northern Ireland has a higher prevalence of rumen fluke compared with England. Carcass characteristics including weight, conformation and fat classification were unaffected by the presence of rumen fluke. Similarly, the DLWG, diarrhea and welfare scores did not differ between infected and non-infected dairy heifers. Furthermore, oxyclozanide treatment reduced rumen fluke FEC, but clinical signs of infection remained low for both groups of heifers. Further research is needed to investigate the production effects due to *C. daubneyi* co-infections with other helminths and in the presence of intercurrent disease.

## Data availability statement

The raw data supporting the conclusions of this article will be made available by the authors, without undue reservation.

## Ethics statement

Ethical review and approval was not required for the animal study because the procedures used in this study were non-invasive and did not require approval from an ethical review body. Written informed consent was obtained from the owners for the participation of their animals in this study.

## Author contributions

AA, BL, MV, MR, KH, and JB conceptualized the study. BL, RM, HE, RH, and KH were responsible for the data collection. EA, AM, and AG performed statistical analysis. JB, EA, AA, NR, and MR wrote the paper. All authors contributed to the article and approved the submitted version.

## Funding

This work was supported by an Industrial Partnership Award (to MR, AA, and JB) from the Biotechnology and Biological Sciences Research Council (BB/N017757/1) with additional financial support from Agrisearch and The Agriculture and Horticulture Development Board, AHDB.

## Conflict of interest

The authors declare that the research was conducted in the absence of any commercial or financial relationships that could be construed as a potential conflict of interest.

## Publisher's note

All claims expressed in this article are solely those of the authors and do not necessarily represent those of their affiliated organizations, or those of the publisher, the editors and the reviewers. Any product that may be evaluated in this article, or claim that may be made by its manufacturer, is not guaranteed or endorsed by the publisher.
